# Supplementary material for: Proliferation to Apoptosis Tumor Cell Ratio as a Biomarker to Improve Clinical Management of Pre-Malignant and Symptomatic Plasma Cell Neoplasms
Source: Int J Mol Sci. 2021 Apr 9;22(8):3895. doi: 10.3390/ijms22083895 (PMC8068942; doi:10.3390/ijms22083895)
Supplement: Supplementary file 1 [file ijms-22-03895-s001.pdf]

Supplementary Material

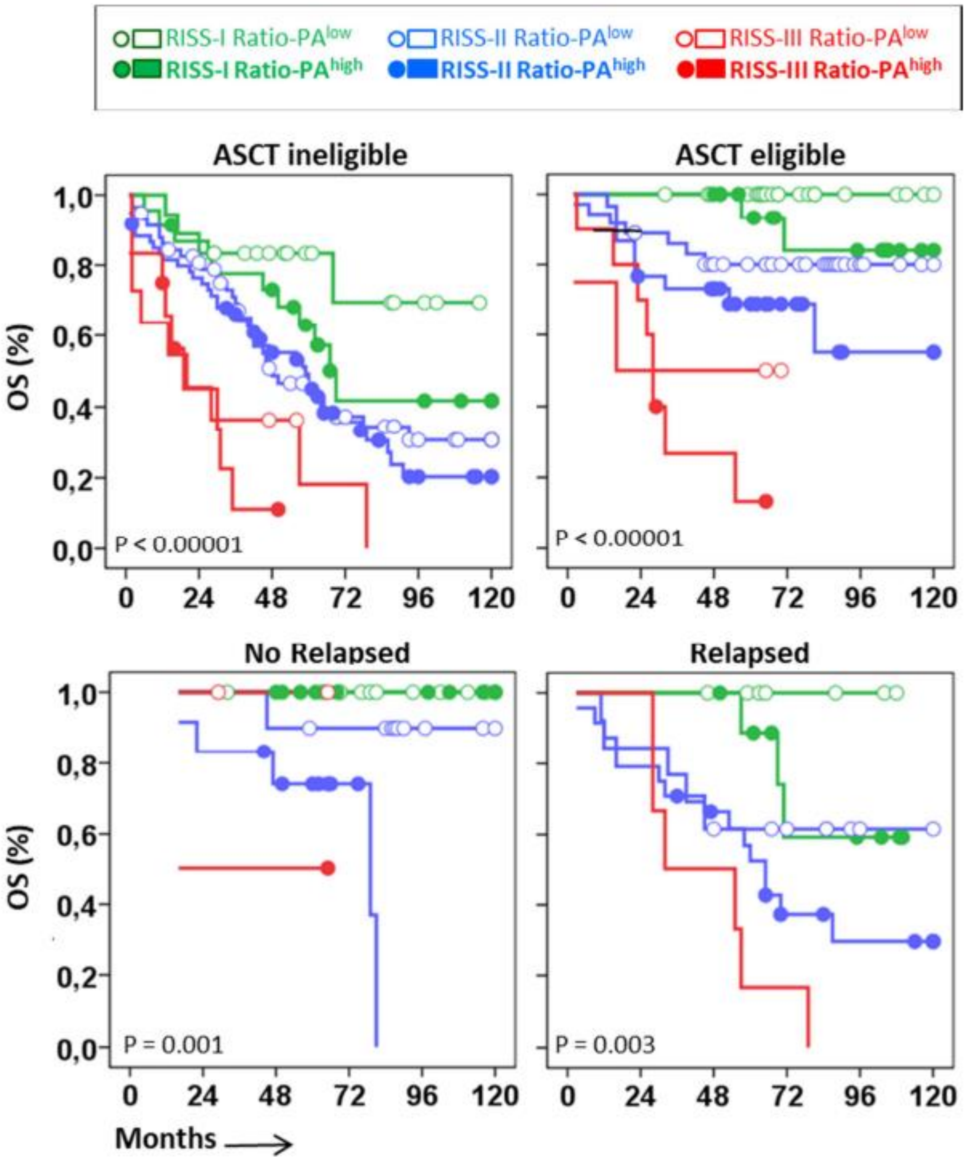

Figure S1. Predictive value of Ratio-PA in plasma cell neoplasm patients requiring treatment. Kaplan-Mejer and Log-rank tests for overall survival (OS) of patients receiving treatment according to the revised international staging system (RISS-I -II and -III), the Ratio-PA (high or low), and the eligibility for autologous stem cell transplantation (ASCT) (upper plots) or relapse after stringent complete response (lower plots).
